# Alexithymia traits outweigh autism traits in the explanation of depression in adults with autism

**DOI:** 10.1038/s41598-021-81696-5

**Published:** 2021-01-26

**Authors:** Carola Bloch, Lana Burghof, Fritz-Georg Lehnhardt, Kai Vogeley, Christine Falter-Wagner

**Affiliations:** 1 Department of Psychiatry and Psychotherapy, University of Cologne, Faculty of Medicine and University Hospital Cologne, Kerpener Str. 62, 50937 Cologne, Germany; 2 Department of Psychiatry and Psychotherapy, Medical Faculty, LMU Munich, Nussbaumstr. 7, 80336 Munich, Germany; 3 Institute of Neuroscience and Medicine (INM3), Research Center Jülich, Leo-Brandt-Str, 52428 Jülich, Germany; 4 Department of Psychology, University of Cologne, Gronewaldstr. 2, 50931 Cologne, Germany

**Keywords:** Medical research, Depression, Autism spectrum disorders

## Abstract

When contemplating the alarming depression rates in adults with autism spectrum disorder (ASD), there is a need to find factors explaining heightened symptoms of depression. Beyond the impact of autism traits, markedly increased levels of alexithymia traits should be considered as a candidate for explaining why individuals with ASD report higher levels of depressive symptoms. Here, we aim to identify the extent to which autism or alexithymia traits indicate depressive symptoms in ASD and whether the pattern of association are specific to ASD. Data of a large (*N* = 400) representative clinical population of adults referred to autism diagnostics have been investigated and split by cases with a confirmed ASD diagnosis (*N* = 281) and cases with a ruled out ASD diagnosis (*N* = 119). Dominance analysis revealed the alexithymia factor, *difficulties in identifying feelings,* as the strongest predictor for depressive symptomatology in ASD, outweighing autism traits and other alexithymia factors. This pattern of prediction was not specific to ASD and was shared by clinical controls from the referral population with a ruled out ASD diagnosis. Thus, the association of alexithymia traits with depression is not unique to ASD and may constitute a general psychopathological mechanism in clinical samples.

## Introduction

### Autism traits and depression

Autism spectrum disorder (ASD) is characterized as a developmental disorder that entails pervasive peculiarities in communication and social interaction, as well as restricted and repetitive behavior, which exist as main diagnostic categories according to ICD-10^1^. Depression remains a prevailing comorbidity in ASD populations. A recent meta-analysis of 29 studies showed a heightened prevalence of current (23%) and lifetime (37%) depression in autistic adults^2^. A subsequent question is whether traits that are inherent to autism suggest a cause for heightened depressive symptoms. It could be assumed that increased autism traits, such as social difficulties and behavioral maladaptation, constitute a possible loading factor. However, mixed empirical findings riddle the literature. For example, a longitudinal study found higher depression scores in children with autism ASD, and higher autism traits predicted higher depression rates in adolescence^3^. In line with that, studies found positive correlations of autism traits with depression in non-clinical samples of adults^4–6^ and a sample of adolescents and adults with ASD^7^. Other studies reported no direct modulation of depressive symptoms by autism traits in adults with ASD^8–10^. Thus, it remains unclear whether autism traits have a direct effect on depression that is not explained by other comorbidities in ASD.

### Alexithymia traits and depression

Besides autism traits as a potential factor for depression in ASD, comorbid factors may account for heightened depressive symptoms. An increasingly discussed psychopathological construct, *alexithymia,* often co-occurs with ASD and is especially interesting in the context of depression in ASD*.* This condition was first described by Sifneos^11^ and is also referred to as *emotional blindness*. In the general population, the estimated prevalence of alexithymia is 10–20%^12–16^. In their recent meta-analysis, Kinnaird et al. reported a markedly increased prevalence of alexithymia in ASD of 50%^17^. Alexithymia measures usually comprise three different traits: *Difficulties in Identifying Feelings* (DIF), *Difficulties Describing Feelings* (DDF), and *Externally-Oriented Thinking* (EOT)^18^. Alexithymia has frequently been linked to depression^19–24^. Regarding the distinct alexithymia subdomains, a meta-analysis of studies found medium-sized effects of *difficulties identifying* and *describing feelings* but only a weak relationship of *externally-oriented thinking* with self-reported depression^20^.

### Alexithymia traits, autism traits, and depression

Considering the high rates of alexithymia in ASD and an association of alexithymia with depression irrespective of ASD, it could be assumed that alexithymia traits modulate depression in ASD. By observing ASD and alexithymia as parallel factors for depression, Fietz and colleagues applied structural equation models on data from an online study addressed to participants from the general population^25^. They showed that autism and alexithymia traits had medium-sized effects on depression. Morie and colleagues conducted an online questionnaire and investigated the effect of autism symptomatology and alexithymia with depressive symptoms in adults with ASD^26^. Their serial mediation analysis provides evidence that autism traits are not directly associated with depression. However, an indirect effect was found, which indicated that autism traits could lead to enhanced depressive symptoms through a positive association with alexithymia and weaker emotion regulation abilities^26^. The validity and generalizability of this online study is limited as the small sample consisted primarily of females (47*f/17*m), and diagnoses were not confirmed by any clinical assessment^26^. Furthermore, the association with depression of both autism and alexithymia raises questions concerning the interpretability of many previous studies testing only one of these predictors or both without controlling their covariation. To date, no study has tested the potentially different effects of alexithymia traits (DIF, DDF, EOT) on depression in a clinical population of individuals with and without ASD, allowing for judgments of specificity.

Consequently, the aims of the current study are to (a) clarify whether autism traits or alexithymia traits better explain depressive symptoms in adults with ASD, (b) establish whether there is a differential predictive power of the three alexithymia traits, and (c) determine whether such predictive associations with depression are specific to ASD. For this purpose, we analyzed data of a representative clinical population of adults referred to outpatient clinics for autism diagnostics. Groups were split into cases with a confirmed ASD diagnosis (*N* = 281) and cases with a ruled out ASD diagnosis (*N* = 119), allowing for direct clinical comparison and statistically accounting for the strong association between autism and alexithymia traits by employing dominance analysis^27^.

## Method

### Participants

Participant characteristics are reported in Table 1. The data analyzed in this study are based on post-hoc naturalistic and comprehensive sampling from the referral population in the outpatient clinic for autism in adulthood at the University Hospital Cologne. Data was collected from 2006 until 2019. The project has been approved by the ethical commission of the medical faculty of the University of Cologne (case number: 20-1432). Data analyzed in this study was fully anonymized. The need to obtain informed consent from the subjects included in this retrospective study has been declared dispensable by the ethics committee of the medical faculty of the University of Cologne. According to §6 para. 1 sentence 1 GDSG NRW (‘Gesundheitsdatenschutzgesetz Nordrhein-Westfalen’) academic staff is allowed to make scientific use of data to which they have access because of their activities, without the consent of the persons concerned being necessary. Individuals were referred to the outpatient clinic for ASD by medical consultants based on suspicion of a possible ASD diagnosis due to reported social-emotional symptoms. Diagnostic procedures throughout the period of data collection were conducted in accordance with German guidelines for the diagnosis of ASD in adulthood^28^, comprising neuropsychological testing and clinical assessment of at least two independent clinicians and a clinical consensus decision. An ASD diagnosis was given if patients met all diagnostic criteria of ICD-10^1^.

**Table 1:** Characteristics of both diagnostic samples: confirmed (ASD+) and ruled out (ASD−) diagnosis of ASD.

Variable	ASD+		ASD−				
	*n*	%	*n*	*%*	*χ*^2^	*df*	*p*
Male	219	78	81	68			
Female	62	22	38	32	3.832	1	0.050
	*M*	*SD*	*M*	*SD*	*t*(398)	*p*	*d*
Age	33.2	11.0	33.5	12.5	0.253	0.801	0.027
IQ	104.3	16.6	100.7	14.7	− 2.055	0.041	− 0.230
PIQ	99.7	16.3	98.0	14.1	− 1.040	0.299	− 0.117
VIQ	107.0	16.4	101.8	15.2	− 2.942	0.003	− 0.326

Number and percentage of males and females in groups with results of Pearson’s chi-square test for frequency distribution in groups. Means (*M*) with standard deviations (*SD*) for metric variables with results of *t*-test (two-tailed, *α* = .05) for independent groups [Levene tests confirmed equal variances (*p* > 0.05)].

The comprehensive clinical population was split into ASD+ cases (*N* = 281), consisting of individuals who received a diagnosis of F84.5 (*n* = 242; *Asperger Syndrome*), F84.1 (*n* = 18; *Atypical Autism*), or F84.0 (*n* = 21; *Childhood Autism*) according to ICD-10^1^, and cases consisting of individuals for whom any F84 diagnosis was ruled out, hence referred to as ASD− (*N* = 119). Thus, the ASD− group comprises individuals who exhibited social difficulties but specifically did not fulfill the criteria for an ASD diagnosis. Patients who received a diagnosis of F84.8 (*n* = 21, *other pervasive developmental disorders*) or F84.9 (*n* = 50, *pervasive developmental disorder—not otherwise specified, PDD-NOS*) were not included in the analyzes because of the ambiguity of group assignment.

Further inclusion criteria were total IQ scores > 70, measured with the German version of the *Wechsler Adult Intelligence Scale* (WIE-III) which includes measures of performance-based IQ (PIQ) and verbal IQ (VIQ)^29^, and less than four missing items on the *Autism-Spectrum Quotient* (AQ)^30^ and the *20-Item Toronto Alexithymia Scale* (TAS-20)^18^. In total, *n* = 19 (ASD+) and *n* = 12 (ASD−) had one missing item, *n* = 5 (ASD+) and *n* = 5 (ASD−) had two missing items, and *n* = 2 (ASD+) had three missing AQ items. For TAS-20, *n* = 8 (ASD+) and *n* = 4 (ASD−) had one missing item, *n* = 2 (ASD+) had two missing items, and *n* = 1 (ASD+) had three missing items. Missing items were replaced by group means. One ASD+ case was not included in the analysis due to an extremely low outlier score on the AQ (< 3 *SD* from *M*).

### Retrieved data

Depressive symptoms were assessed with the German version of the *Beck Depression Inventory* (BDI)^31^. BDI is a self-report questionnaire that assesses depressive symptoms over 21 items^32^. Each item was answered on a four-point scale, scoring from 0–3, with higher scores indicating increased symptom severity. Scores in the range of 11–17 indicate mild to moderate depressive symptoms and scores ≥ 18 classify clinically relevant depression^31^. In the ASD+ and ASD− groups, 25.27% and 20.17% of cases reported mild-moderate symptoms, respectively. In the ASD+ and ASD− groups, 30.60% and 41.18% reported clinically relevant depressive symptoms, respectively.

The AQ is a well-established 50-item self-report questionnaire assessing autism traits including *social skills*, *difficulties in attention switching*, the degree of *attention to detail*, and difficulties in *communication* and *imagination*^30^. Each of the 50 items was answered on a Likert-scale, ranging from 1 = *agree* to 4 = *disagree* and re-coded to 1 or 0 for summing. Scores > 32 depict pronounced autism traits^30^. In the ASD+ and ASD− groups, 83.63% and 78.15% of cases reported autism traits above this threshold, respectively. The internal consistency of the scale was good (α = 0.86).

The TAS-20 is a self-report questionnaire assessing alexithymia on three dimensions (DIF, DDF, and EOT)^18^. Each item was answered on a five-point Likert-scale, ranging from 5 = *strongly agree* to 1 = *strongly disagree*. The recommended cut-off for clinically relevant alexithymia is 61^33^. In the ASD+ and the ASD− samples, 66.19% and 68.91% reported clinically relevant alexithymia traits, respectively. Cronbach’s alphas for all subscales revealed good internal consistencies for DIF (*α* = 0.85) and DDF (*α* = 0.74) and acceptable internal consistency for EOT (*α* = 0.60).

### Statistical procedures

Data preprocessing and analysis were conducted in RStudio^34^ using the statistical programming language R^35^. Based on their extensive taxometric analysis, Parker et al. argued that the latent structure of alexithymia should be considered continuous rather than categorical^36^. Accordingly, we analyzed alexithymia traits based on metric scales. Two sample *t*-tests were conducted to compare ASD+ and ASD− in terms of TAS-20, AQ, and BDI. Pearson zero-order correlations measures of interest were calculated with Bonferroni adjustment (Table 2). For multivariate analysis, regression models were calculated with autism traits (AQ) and alexithymia traits (DIF, DDF, EOT) as predictors of self-reported depressive symptoms (BDI) in ASD+ and ASD− samples. The samples differed significantly in IQ scores, with higher scores in the ASD+ sample (*t*(398) =  − 2.055, *p* < 0.05, *d* =  − 0.230), driven by higher scores in VIQ (*t*(398) =  − 2.942, *p* < 0.05, *d* =  − 0.326). We included age, sex, and measures of PIQ, and VIQ as control variables to account for potential confounds (see Table 1 for summary statistics and significance tests). The model for the ASD− sample met all assumptions for general linear models; namely, sample sizes were sufficient for the number of predictors, adequate model fit (< 5% of standardized residuals > 2), independence of errors (Durbin–Watson statistic ≈ 2), lack of multicollinearity (variance inflation factor < 2 for all predictors in all models), and homoscedasticity for the ASD− model. As for the model of the ASD+ sample, heteroscedasticity was implied by funneled residual plots and a significant result in the Breusch Pagan Test, robust regression with heteroscedasticity-consistent covariance matrix (HCCM) was conducted, using the *sandwich*^37^ and *lmtest*^38^ packages in R. HCCMs were retrieved by the *vcovHC()* function, applying the HC3 method based on recommendations by Long & Ervin^39^. HCCM was proposed as an adequate method for adjustment of errors in case of heteroscedasticity by Rosopa et al.^40^.

**Table 2:** Descriptive statistics and correlations for variables of interest.

Variable	Sample	*M*	*SD*	BDI	AQ	DIF	DDF
BDI	ASD+	13.9	10.4	–			
	ASD−	15.7	10.5				
AQ	ASD+	38.7	6.1	0.25 (0.000)	–		
	ASD−	37.6	5.8	0.12 (0.189)			
DIF	ASD+	22.1	6.1	0.41 (0.000)	0.52 (.000)	–	
	ASD−	23.2	6.6	0.25 (0.006)	0.44 (0.000)		
DDF	ASD+	19.1	3.8	0.26 (0.000)	0.47 (0.000)	0.59 (0.000)	–
	ASD−	18.9	4.0	− 0.01 (0.877)	0.47 (0.000)	0.61 (0.000)	
EOT	ASD+	23.4	5.2	− 0.03 (0.643)	0.00 (0.985)	0.08 (0.203)	0.22 (0.000)
	ASD−	23.7	5.1	0.06 (0.551)	0.02 (0.830)	− 0.01 (0.923)	0.17 (0.065)

Pearson’s product moment correlation coefficients (*p* values, Bonferroni-adjusted).

Correlations of autism and alexithymia traits could have problematic results when interpreting their relative effects through beta coefficients. Beta coefficients represent the total effects, whereas general dominance weights (GDW) constitute a global index of importance. This was suggested as a new comparison standard for multiple regression because it considers direct, total, and partial effects^27^. This method was previously introduced regarding the inference of linear effects of alexithymia and ASD on empathy^41^. *GDW*s were an adequate and easily interpretable effect size for answering the research questions concerning the relative effects of alexithymia and autism traits in our analysis. *GDW*s were calculated with their bootstrapped 95% confidence intervals (100 resamples) using the *yhat* package in R^42^. *GDW*s were retrieved by dominance analysis^43^, in which unique variance explained by each predictor was calculated by the squared semipartial correlation averaged across all models in all possible subsets that included that predictor^27^. *GDW*s together sum up to the total determination factor *R*^2^ of the model. Therefore, they allowed for the calculation of the relative proportion of each predictor in variance explanation and, thus, the ranking of predictors within the models^44^.

## Results

Table 2 depicts mean scores and correlations of the measures of interest in the groups. The ASD+ sample did not significantly differ from the ASD− sample in any TAS-20 subdomains, DIF (*t*(398) = 1.698, *p* = 0.090, *d* = 0.183), DDF (*t*(398) =  − 0.555, *p* = 0.579, *d* =  − 0.060), and EOT (*t*(398) = 0.488, *p* = 0.626, *d* = 0.054). The groups did not differ significantly in their levels of AQ (*t*(398) =  − 1.693, *p* = 0.091, *d* =  − 0.187) and BDI (*t*(398) = 1.579, *p* = 0.115, *d* = 0.172).

Regarding correlations with depressive symptoms in the ASD + sample, BDI scores significantly increased with AQ (*r* = 0.25, *p* < 0.001, 95% *CI* [0.13, 0.35]), DIF (*r* = 0.41, *p* < 0.001, 95% *CI* [0.31, 0.51]), and DDF (*r* = 0.26, *p* < 0.001, 95% *CI* [0.15, 0.37]), but not with EOT (*r* =  − 0.03, *p* = 0.643, 95% *CI* [ − 0.14, 0.09]). In the ASD− sample, BDI significantly increased with DIF (*r* = 0.25, *p* < 0.05, 95% *CI* [0.08, 0.41]), but not with AQ (*r* =  0.12, *p* = 0.189, 95% *CI* [ − 0.06, 0.29]), DDF (*r* =  − 0.01, *p* < 0.877, 95% *CI* [ − 0.19, 0.16]) or EOT (*r* = 0.06, *p* = 0.551, 95% *CI* [ − 0.13, 0.23]). Considering correlations of autism and alexithymia traits in the ASD + sample, DIF significantly increased with AQ (*r* = 0.52, *p* < 0.001, 95% *CI* [0.43, 0.60]) and DDF (*r* = 0.47, *p* < 0.001, 95% *CI* [0.38, 0.56]). Similarly, AQ significantly increased with DIF (*r* = 0.44, *p* < 0.001, 95% *CI* [0.28, 0.57]) and DDF (*r* = 0.47, *p* < 0.001, 95% *CI* [0.32, 0.60]) in the ASD− sample. EOT was not correlated with AQ in either sample (ASD + : *r* =   0.00, *p* = 0.985, 95% *CI* [ − 0.12, 0.12]/ASD−: *r* = 0.02, *p* = 0.830, 95% *CI* [ − 0.16, 0.20]).

Table 3 depicts regression estimates and results of dominance analysis with BDI as the dependent variable. The model in the ASD + sample explained 23.2% variance in BDI score (*F*(8,272) = 10.27, *p* < 0.001). *GDW* estimates of AQ, DIF, DDF, and EOT allowed for ranking predictors in terms of importance by their proportion of variance explanation (*R*^*2*^). In the ASD + sample, DIF stood out as the strongest predictor (*GDW* = 0.116, CI [0.062, 0.173], 50.0% of *R*^*2*^), DDF was the second (*GDW* = 0.029, CI [0.014, 0.059], 12.5% of *R*^*2*^), and AQ the third strongest predictor (*GDW* = 0.020, CI [0.009, 0.049], 8.6% of *R*^*2*^) with negative coefficients. This indicates that autism traits did not affect depression beyond alexithymia traits. The model in the ASD− sample explained 18.3% variance of BDI scores (*F*(8,110) = 3.08, *p* < 0.01). Estimates revealed a similar structure as the ASD + sample: DIF was the strongest predictor (*GDW* = 0.068, CI [0.012, 0.144], 37.1% of *R*^*2*^), DDF was ranked second (*GDW* = 0.024, CI [0.005, 0.114], 13.1% of *R*^*2*^), and AQ third (*GDW* = 0.019, CI [0.002, 0.078], 10.4% of *R*^*2*^). EOT was the weakest predictor of depressive symptoms with small effect sizes in the ASD+ sample (*GDW* = 0.003, CI [0.001, 0.037], 1.3% of *R*^*2*^) and in the ASD− sample (*GDW* = 0.002, CI [0.001, 0.049], 1.1% of *R*^*2*^).

**Table 3:** Models with BDI as dependent variable.

Predictors	ASD+				ASD−			
	*β*	*SE*_*adj*_	GDW	LL, UL	*β*	SE	GDW	LL, UL
Age	0.184	0.061	0.042	0.007, 0.094	0.013	0.079	0.003	0.001, 0.042
Sex	0.147	1.294	0.016	0.003, 0.044	0.164	2.067	0.015	0.001, 0.085
PIQ	0.030	0.043	0.004	0.001, 0.020	− 0.061	0.080	0.020	0.002, 0.080
VIQ	− 0.038	0.045	0.002	0.001, 0.015	− 0.199	0.077	0.032	0.004, 0.121
AQ	− 0.006	0.137	0.020	0.009, 0.049	0.195	0.199	0.019	0.002, 0.078
**DIF**	**0**.**369**	**0**.**136**	**0**.**116**	**0**.**062**,** 0**.**173**	**0**.**354**	**0**.**187**	**0**.**068**	**0**.**012**,** 0**.**144**
DDF	0.064	0.194	0.029	0.014, 0.059	− 0.302	0.313	0.024	0.005, 0.114
EOT	− 0.079	0.129	0.003	0.001, 0.037	0.017	0.197	0.002	0.001, 0.049
*R*^*2*^			0.232				0.183	

Models for ASD+ (*N* = 281) and ASD− (*N* = 119) samples. Standardized regression coefficients (*β*) with standard errors (*SE*) or HCCM-adjusted errors (*SE*_*adj*_). General dominance weights (*GDW*) retrieved by dominance analysis with lower limits (*LL*) and upper limits (*UL*) of bootstrapped confidence intervals. Strongest predictor in each model in bold.

## Discussion

The key findings of the current study are:

- Alexithymia traits (i.e. DIF & DDF) outweigh autism traits in the multivariate prediction of depressive symptoms in adults with ASD,
- The alexithymia trait, *difficulties identifying feelings,* is the strongest predictor of depressive symptom severity, and
- Similar patterns of prediction in both models, for adults with and without ASD, imply a general mechanism for depression in clinical samples.

Autism traits significantly correlated with depressive symptoms, which is in line with studies that also reported positive correlations^4–7^. Nevertheless, considering the high correlation of alexithymia and autism traits, our multivariate analysis puts this association in a new perspective because autism traits were only ranked third after alexithymia traits *difficulties identifying feelings* and *difficulties describing feelings*. This aligns with past studies, particularly with the findings of Morie et al.^26^, as they resemble the direct effect of alexithymia on depressive symptoms in adults with ASD. Beyond replication, our results imply *difficulties identifying feelings* as the main factor in predicting depressive symptom severity. Furthermore, the effect of *difficulties identifying feelings* is in accordance with Li et al. who reported an overall medium effect size in their meta-analysis of studies from the general population and depression patients^20^. Li et al. further reported medium effect sizes for *difficulties describing feelings* on depression, whereas we found only small effect sizes in the multivariate observation. As univariate correlations revealed a positive correlation with self-reported depressive symptoms, the small effect in the dominance analysis could hint at an intersection of variance explanation with autism traits. As AQ already includes communication difficulties, it may act as a suppressor for the scale assessing *difficulties describing feelings*.

Importantly, we need to understand whether prediction patterns of depression are unique to ASD for clinical decision making with respect to treatment planning. We found depressive symptoms to be similarly modulated by alexithymia traits and specifically explained by *difficulties identifying feelings* in a clinical population with a ruled out ASD diagnosis. Hence, alexithymia, in general, and *difficulties identifying feelings,* in particular, might be generally associated with depression requiring attention in treatment planning beyond ASD.

There are some important methodological and theoretical implications of this study. First, our results support the suggestion that the alexithymia subdomains should be considered individually to make inferences about distinct and subtle effects^20,24,45^. As the subdomains showed different temporal stability^24^, dissimilar internal consistencies^13,33,46^, may distinctively relate to measures of psychological stress^47^, and are semantically different^20,45^. Thus, observations of individual subdomains should be standard. Subdomain splitting makes sense with regard to the different extent of social characteristics comprising the subdomains, especially in the context of ASD. While *difficulties in identifying feelings* and *externally-oriented thinking* are person-level domains, *difficulties describing feelings* represents a social domain of alexithymia. Thus, for individuals with pronounced autism traits comprising social communication difficulties, high scores in this domain might reflect a trend of social withdrawal rather than a cause of malfunctioning emotional access.

Another important aspect of our study is the replication of prior results that showed high correlations of autism and alexithymia traits^41,48^. We encourage future research to consider this finding in their methodological procedures. For relative effects in the case of covariation of predictors, dominance weights demonstrate a reasonable alternative to beta coefficients as they represent a valid and easily interpretable estimate of *relative* importance of each predictor^27,44^.

It has been suggested by different authors that alexithymia could be used to define subgroups within the autism spectrum^12,49^. Based on our results, we would tentatively agree but additionally argue that alexithymia should be regarded in terms of subdomains and as continuous variables^36^. Our results demonstrate that a subgroup of adults with ASD can be described by high levels of *difficulties identifying feelings,* and this subgroup may specifically profit from interventions that target emotional regulation abilities and foster introspection. Regarding this potential subgroup in ASD, it could be assumed that heightened rates of *difficulties identifying feelings* have their origin outside autism features. Szatmari et al. found that parents of children with ASD reported significantly higher degrees of *difficulties identifying feelings* compared to parents of children with Prader Willi syndrome^50^. This result was recently replicated with the addition that *difficulties identifying feelings* were associated with depression in mothers and fathers of children with ASD^51^. As studies showed that alexithymia is potentially passed on to children through heredity^52^ and family interactions^53^, it could be suggested that heightened rates of *difficulties in identifying feelings* in a subgroup could be explained by external factors (family or affiliated persons). Likewise, it could be suggested that autism traits are potentially heightened within the family, which gives rise to alexithymia. Etiological pathways remain unclear, and future studies should be conducted that specifically target alexithymia in individuals with ASD and their caregivers in a longitudinal design. Such research could better inform the possible origins of pronounced alexithymia traits in ASD and allow for inference of alexithymia as a cause for, or consequence of, depression in ASD.

Aside from the methodological advantages and important implications, the current study has limitations. First of all, we applied a retrospective cross-sectional design that does not allow for causal inference. Questions about the directionality of effects and etiological pathways remain a target for future prospective longitudinal studies. Additionally, our results are based on post-hoc naturalistic and comprehensive sampling from the referral population in two outpatient clinics for adults with autism. While this approach has the asset of delivering directly translatable generalization into the clinical reality of adult outpatient clinics, the clinical comparison group of individuals with suspicion of, but ultimately ruled-out, ASD diagnosis results in a heterogeneous clinical sample. Future studies will need to add to the question of generalizability by testing homogeneous clinical samples characterized by, for instance, social phobia. Another limitation that should be considered is that all measures were retrieved via self-report. Deploying questionnaires for all measures potentially results in variance that is only due to this common method. Future studies should include various methods to compensate for this bias.

By deploying adequate methods for inference about the differential predictive power of alexithymia and autism traits in a large representative referral population for autism diagnostics in adulthood, the current results suggest that the alexithymia trait, *difficulties identifying feelings,* acts as a major predictor for depression in adults with ASD. Our results further show that this effect is not specific to ASD and potentially reflects a general mechanism in clinical samples.

## Additional information

The use of language in this article was chosen based on suggestions in: Tepest, R. (2020). The meaning of diagnosis for different designations in talking about autism. Journal of Autism and Development. (DOI: 10.1007/s10803-020-04584-3).

## Data Availability

The aggregated data analyzed during the current study and the R Script used for analysis are available from the corresponding author on reasonable request.
