# Author Correction: Alexithymia traits outweigh autism traits in the explanation of depression in adults with autism

**DOI:** 10.1038/s41598-021-98757-4

**Published:** 2021-09-17

**Authors:** Carola Bloch, Lana Burghof, Fritz-Georg Lehnhardt, Kai Vogeley, Christine Falter-Wagner

**Affiliations:** 1 Department of Psychiatry and Psychotherapy, University of Cologne, Faculty of Medicine and University Hospital Cologne, Kerpener Str. 62, 50937 Cologne, Germany; 2 Department of Psychiatry and Psychotherapy, Medical Faculty, LMU Munich, Nussbaumstr. 7, 80336 Munich, Germany; 3 Institute of Neuroscience and Medicine (INM3), Research Center Jülich, Leo-Brandt-Str, 52428 Jülich, Germany; 4 Department of Psychology, University of Cologne, Gronewaldstr. 2, 50931 Cologne, Germany

Correction to: *Scientific Reports* 10.1038/s41598-021-81696-5, published online 26 January 2021

The original version of this Article contained errors, that affected the descriptive statistics. Inferential statistics remained unaffected.

In Table 1, the Cohen’s d values were incorrectly given. The correct and incorrect values appear below.

Incorrect:

**Table:** 

	*d*
Age	0.057
IQ	− 0.212
PIQ	− 0.107
VIQ	− 0.314

Correct:

**Table:** 

	*d*
Age	0.027
IQ	− 0.230
PIQ	− 0.117
VIQ	− 0.326

As a result, in the Method section, under the subheading ‘Statistical procedures’,

“The samples differed significantly in IQ scores, with higher scores in the ASD+ sample (*t*(398) = − 2.055, *p* < 0.05, *d* = − 0.212), driven by higher scores in VIQ (*t*(398) = − 2.942, *p* < 0.05, *d* = − 0.314).”

now reads:

“The samples differed significantly in IQ scores, with higher scores in the ASD+ sample (*t*(398) = − 2.055, *p* < 0.05, *d* = − 0.230), driven by higher scores in VIQ (*t*(398) = − 2.942, *p* < 0.05, *d* = − 0.326).”

In the Results section,

“The ASD+ sample did not significantly differ from the ASD− sample in any TAS-20 subdomains, DIF (*t*(398) = 1.698, *p* = 0.090, *d* = 0.186), DDF (*t*(398) = − 0.555, *p* = 0.579, *d* = − 0.061), and EOT (*t*(398) = 0.488, *p* = 0.626, *d* = 0.053). The groups did not differ significantly in their levels of AQ (*t*(398) = − 1.693, *p* = 0.091, *d* = − 0.185) and BDI (*t*(398) = 1.579, *p* = 0.115, *d* = 0.173).”

now reads:

“The ASD+ sample did not significantly differ from the ASD− sample in any TAS-20 subdomains, DIF (*t*(398) = 1.698, *p* = 0.090, *d* = 0.183), DDF (*t*(398) = − 0.555, *p* = 0.579, *d* = − 0.060), and EOT (*t*(398) = 0.488, *p* = 0.626, *d* = 0.054). The groups did not differ significantly in their levels of AQ (*t*(398) = − 1.693, *p* = 0.091, *d* = − 0.187) and BDI (*t*(398) = 1.579, *p* = 0.115, *d* = 0.172).”

Furthermore, in Table 2, the BDI values for the Variables “AQ” sample “ASD − ”, “EOT” sample “ASD + ”, and “EOT” sample “ASD − ” were incorrect. Additionally, the AQ values for the Variables “DDF” sample “ASD − ”, “EOT” sample “ASD + ”, and “EOT” sample “ASD − ” were incorrect. The correct and incorrect values appear below.

Incorrect:

**Table:** 

Variable	Sample	BDI	AQ
AQ	ASD +	0.25 (0.000)	–
	ASD −	0.20 (0.033)	
DDF	ASD +	0.26 (0.000)	0.47 (0.000)
	ASD −	− 0.01 (0.877)	0.40 (0.000)
EOT	ASD +	− 0.03 (0.650)	− 0.01 (0.827)
	ASD −	0.05 (0.551)	0.00 (0.952)

Correct:

**Table:** 

Variable	Sample	BDI	AQ
AQ	ASD +	0.25 (0.000)	–
	ASD −	0.12 (0.189)	
DDF	ASD +	0.26 (0.000)	0.47 (0.000)
	ASD −	− 0.01 (0.877)	0.47 (0.000)
EOT	ASD +	− 0.03 (0.643)	0.00 (0.985)
	ASD −	0.06 (0.551)	0.02 (0.830)

As a result, in the Results section,

“Regarding correlations with depressive symptoms in the ASD + sample, BDI scores significantly increased with AQ (*r* = 0.25, *p* < 0.001, 95% *CI* [0.13, 0.35]), DIF (*r* = 0.41, *p* < 0.001, 95% *CI* [0.31, 0.51]), and DDF (*r* = 0.26, *p* < 0.001, 95% *CI* [0.15, 0.37]), but not with EOT (*r* = − 0.03, *p* = 0.650, 95% *CI* [ − 0.14, 0.09]). In the ASD− sample, BDI significantly increased with AQ (*r* = 0.20, *p* = < 0.05, 95% *CI* [0.02, 0.36]), and with DIF (*r* = 0.25, *p* < 0.05, 95% *CI* [0.08, 0.41]), but not with DDF (*r* = − 0.01, *p* < 0.877, 95% *CI* [ − 0.19, 0.16]) or EOT (*r* = 0.05, *p* = 0.551, 95% *CI* [ − 0.13, 0.23]). Considering correlations of autism and alexithymia traits in the ASD + sample, DIF significantly increased with AQ (*r* = 0.52, *p* < 0.001, 95% *CI* [0.42, 0.60]) and DDF (*r* = 0.47, *p* < 0.001, 95% *CI* [0.37, 0.56]). Similarly, AQ significantly increased with DIF (*r* = 0.44, *p* < 0.001, 95% *CI* [0.28, 0.58]) and DDF (*r* = 0.40, *p* < 0.001, 95% *CI* [0.24, 0.54]) in the ASD− sample. EOT was not correlated with AQ in either sample (ASD + : *r* = − 0.01, *p* = 0.827, 95% *CI* [ − 0.12, 0.10]/ASD−: *r* = 0.00, *p* = 0.952, 95% *CI* [ − 0.18, 0.18]).”

now reads:

“Regarding correlations with depressive symptoms in the ASD + sample, BDI scores significantly increased with AQ (*r* = 0.25, *p* < 0.001, 95% *CI* [0.13, 0.35]), DIF (*r* = 0.41, *p* < 0.001, 95% *CI* [0.31, 0.51]), and DDF (*r* = 0.26, *p* < 0.001, 95% *CI* [0.15, 0.37]), but not with EOT (*r* = − 0.03, *p* = 0.643, 95% *CI* [ − 0.14, 0.09]). In the ASD− sample, BDI significantly increased with DIF (*r* = 0.25, *p* < 0.05, 95% *CI* [0.08, 0.41]), but not with AQ (*r* = 0.12, *p* = 0.189, 95% *CI* [ − 0.06, 0.29]), DDF (*r* = − 0.01, *p* < 0.877, 95% *CI* [ − 0.19, 0.16]) or EOT (*r* = 0.06, *p* = 0.551, 95% *CI* [ − 0.13, 0.23]). Considering correlations of autism and alexithymia traits in the ASD + sample, DIF significantly increased with AQ (*r* = 0.52, *p* < 0.001, 95% *CI* [0.43, 0.60]) and DDF (*r* = 0.47, *p* < 0.001, 95% *CI* [0.38, 0.56]). Similarly, AQ significantly increased with DIF (*r* = 0.44, *p* < 0.001, 95% *CI* [0.28, 0.57]) and DDF (*r* = 0.47, *p* < 0.001, 95% *CI* [0.32, 0.60]) in the ASD− sample. EOT was not correlated with AQ in either sample (ASD + : *r* = 0.00, *p* = 0.985, 95% *CI* [ − 0.12, 0.12]/ASD−: *r* = 0.02, *p* = 0.830, 95% *CI* [ − 0.16, 0.20]).”

The original Article has been corrected.

